# T lymphocytes among HIV-infected and -uninfected infants: CD4/CD8 ratio as a potential tool in diagnosis of infection in infants under the age of 2 years

**DOI:** 10.1186/1479-5876-3-6

**Published:** 2005-02-01

**Authors:** Lynn S Zijenah, David A Katzenstein, Kusum J Nathoo, Simbarashe Rusakaniko, Ocean Tobaiwa, Christine Gwanzura, Arsene Bikoue, Margaret Nhembe, Petronella Matibe, George Janossy

**Affiliations:** 1 Department of Immunology, College of Health Sciences University of Zimbabwe, Harare, Zimbabwe; 2 Division of Infectious Diseases and AIDS Research, Stanford University Medical School, Stanford, California, USA; 3 Department of Paediatrics, College of Health Sciences University of Zimbabwe, Harare, Zimbabwe; 4 Department of Community Medicine, College of Health Sciences University of Zimbabwe, Harare, Zimbabwe; 5 Department of Haematology, College of Health Sciences University of Zimbabwe, Harare, Zimbabwe; 6 Department of Immunology and Molecular Pathology, Royal Free and University College Medical School, London, UK; 7 Department of Flow Cytometry, MFN International, Asmara, Eritrea

## Abstract

**Background:**

Serologic tests for HIV infection in infants less than 18 months do not differentiate exposure and infection since maternally acquired IgG antibodies may be detected in infants. Thus, the gold standard for diagnosis of HIV-1 infection in infants under the age of 2 years is DNA or reverse transcriptase polymerase chain reaction. There is an urgent need to evaluate alternative and cost effective laboratory methods for early diagnosis of infant HIV-1 infection as well as identifying infected infants who may benefit from cotrimoxazole prophylaxis and/or initiation of highly active antiretroviral therapy.

**Methods:**

Whole blood was collected in EDTA from 137 infants aged 0 to 18 months. DNA polymerase chain reaction was used as the reference standard for diagnosis of HIV-1 infection. T-cell subset profiles were determined by flow cytometry.

**Results:**

***Seventy-six ***infants were DNA PCR positive while 61 were negative. The median CD4 counts of PCR negative infants were significantly higher than those of the PCR positive infants, *p < 0.001*. The median CD4/CD8 ratio and the %CD4 of the PCR positive infants were both significantly lower than those of the negative infants, *p < 0.001*. The CD4/CD8 ratio had a >98% sensitivity for diagnosis of HIV-1 infection and a specificity of >98%.

**Conclusion:**

The CD4/CD8 ratio appears useful in identifying HIV-infected infants. The development of lower cost and more robust flow cytometric methods that provide both CD4/CD8 ratio and %CD4 may be cost-effective for HIV-1 diagnosis and identification of infants for cotrimoxazole prophylaxis and/or highly active antiretroviral therapy.

## Background

Endemic HIV infection, in sub-Saharan Africa, where in many countries more than 20% of pregnant women are HIV seropositive leads to a diagnostic problem in the evaluation of their infants. Without intervention, more than 25% of infants born to seropositive women will acquire HIV infection in the first year of life. HIV testing with enzyme immunoassay-based rapid tests have expanded capacity to identify seropositive women and provide interventions, but even with single dose Nevirapine and other antiretrovirals, infection of infants still exceeds 10% in the first year of life. Serologic tests for HIV do not accurately identify those infants who have acquired infection within the first 18 months of life because of transplacentally acquired maternal IgG antibodies. As antimicrobial and antiviral interventions are developed to reduce morbidity and mortality, among infants born to seropositive women, the early diagnosis of infection is increasingly important. The gold standard for diagnosis of HIV-1 infection in infants under the age of 2 years is DNA polymerase chain reaction (PCR) or reverse transcriptase (RT)-PCR [1-3]. With the increasing availability of single dose nevirapine for prevention of mother-to-child transmission (MTCT) of HIV [4] and generic antiretroviral drugs for treatment of AIDS in resource-poor countries, there is an urgent need to develop alternative laboratory methods for early diagnosis of infant HIV-1 infection as well as identifying infants who meet the criteria for commencing cotrimoxazole prophylaxis and/or initiation of ARV therapy.

The World Health Organization (WHO) recommends initiation of highly active antiretroviral therapy (HAART) in HIV-seropositive infants under the age of 18 months, who have WHO Pediatric Stage III disease and CD4 percentage (%CD4) <20% in resource-poor countries where %CD4 are available but virologic tests (DNA PCR, RT-PCR or immune-complex dissociated p24 antigen) for confirmation of HIV infection are not available [5]. Thus, in infants under the age of 18 months, a single laboratory test that can identify both HIV-infection and provide %CD4, two important parameters for decision-making in initiating HAART, may be useful. Infants who are positively identified as HIV-infected and meet clinical criteria are likely to benefit from HAART.

Immunological changes in HIV-1 infection include a decrease in CD4^+ ^cells, a transient increase in CD8^+ ^cells, total lymphocytes and inversion of the CD4/CD8 ratio [6,7]. As HIV infection progresses, the CD4^+ ^cells decline, while the CD8^+ ^cells which may remain at high levels for long periods, eventually decrease but not to baseline levels. Since in healthy children the CD4^+ ^and CD8^+ ^cells account for 60% and 30% of the T lymphocytes respectively, a normal CD4/CD8 ratio should always be >1.0. Thus, in HIV-1 infection where there is a decrease in CD4^+ ^cells and an increase in CD8^+ ^cells, the reversal of the CD4/CD8 to <1.0 should in theory be useful for diagnosis of HIV-1 infection.

Certain flow cytometers when used in tandem with a haematological analyzer can provide absolute CD4^+ ^cell counts, their percentages as well as the CD4/CD8 ratio. The use of the ratio in combination with %CD4, may lead to a timely identification of infected infants, who meet the WHO criteria for initiation of CTX prophylaxis and/or ARV therapy. While the infrastructure needed to conduct flow-cytometric analyses of HIV infection are still largely confined to a few centers, an increasing number of point of care diagnostic testing systems, inexpensive methods to measure CD4 cells are currently in development. These include the development and evaluation of simplified volumetric flow cytometric methods using a low cost flow cytometer that can be powered from a car battery or by solar panels (Cyflow SL, Partec, Munster, Germany) by Cassens and colleagues [8,9] and modification of a commercially available 4-parameter flow cytometer, Luminex 100 (Luminex, Austin Texas, USA) to a compact portable prototype instrument that can operate with a 12-volt rechargeable battery [10]. Furthermore, use of generic CD4, CD8 and CD45 fluorescence-conjugated monoclonal antibodies can reduce the cost of determining T cell subset profile even when employing standard flow cytometers [10,11].

The main objective of the current study was to evaluate the CD4/CD8 ratio for diagnosis of subtype C HIV-1 infection in infants under the age of 2 years among infants where DNA PCR was performed to diagnose HIV infection.

## Materials and Methods

### Study cohort

The infant specimens used in this study were obtained from two independent prospective studies; the short course zidovudine (AZT) and Pediatric AIDS Clinical definition (PACD). The Medical Research Council and the Institutional Ethics Committees approved both studies and present study. The infants included in the study were under the age of 2 years. All the infants were breastfeeding at the time of specimen collection.

Infants who were followed in the short course AZT study aimed at preventing MTCT were enrolled between May 2001 and June 2002. In the AZT study, mothers received short course AZT starting at 36 weeks gestation and throughout labor. Their infants received AZT for 7 days.

In the PACD study, hospitalized children aged between 2 months and 18 months were prospectively enrolled into the study, between July 2002 and July 2003, following informed consent from their mothers. The mothers of these children did not receive antiretroviral therapy for prevention of MTCT nor for HIV disease. The PACD study is a hospital based analytical cross-sectional survey. Laboratory specimens were obtained once from each subject. Children presenting in the moribund state, requiring immediate resuscitation or those with known HIV status or those whose mothers/guardians refused to sign the informed consent were excluded from the study. The objective of the PACD study is to identify clinical symptoms, associated with HIV-1 infection in infants under the age of 18 months, which may be used in the absence of laboratory tests, for HIV-1 diagnosis.

### Blood collection and processing

A total volume of 2 ml whole blood was collected in ethylenediamine tetraacetic acid from each of the 137 infants in the short course AZT and the PACD studies. The whole blood was then aliquoted into two tubes (500 microlitres in each) for determination of T cell subset profiles, and the second for DNA PCR and the rest centrifuged at 200 g to obtain plasma, which was stored at -80°C.

The laboratory tests described below were conducted in a blind fashion.

### Flow cytometry analysis

T cell subset profiles were determined by flow cytometry using a Coulter Epics XL equipped with System II software (Beckman Coulter, Miami, Florida, USA) within 4 hours of blood collection. This flow cytometer was run, in a double platform setting where the absolute counts for both white blood cells and lymphocytes were obtained on a Celldyn 3500R haematological analyzer (Abbott, GmbH, Germany). Then from the combined results, the absolute CD4^+ ^and CD8^+ ^cell counts, CD4/CD8 ratios, as well as the %CD4 and %CD8 values among lymphocytes were automatically calculated.

### DNA PCR analysis

DNA PCR Roche amplification assay version 1.5 (Roche Diagnostics, Branchburg, NJ, USA) was employed as the reference standard for diagnosis of infant HIV-1 infection status. DNA extraction, amplification and detection were performed and results interpreted following the manufacturer's instructions (Roche Diagnostics, Branchburg, NJ, USA) as we previously described [12,13].

### Infant grouping

Reference ranges of T cell subset profiles for infants and children are usually stratified by age as <12 months, 1 to 5 years and 6–12 years. In the current study, in addition to overall evaluation of samples obtained from all the infants aged 0 to 18 months, the evaluated parameters were also compared based on infant age groups 0–11, and 12–18 months.

### Statistical Analysis

Diagnostic tests (sensitivity, specificity, positive predictive value (PPV), negative predictive value (NPV) and test efficiency (TE) with 95% confidence interval) were used to assess the assay under evaluation with DNA PCR as the reference standard. A MTCT prevalence rate of 30.7% (based on the current rate in Zimbabwe) was used in the calculations of PPV and NPV. Sensitivity was defined as the percentage of reference standard HIV positive samples found reactive by assay under evaluation. Specificity was defined as the percentage of reference standard HIV-negative samples that were negative by the assay under evaluation. TE refers to the overall ability of a test to correctly identify all positives and negatives (the absence of false positives and false negatives). It is a combination of the sensitivity and the specificity of an assay and gives an idea of the total effectiveness of the assay. PPV was defined as the probability that a specimen had CD4:CD8 ratio less than 1 when the test was DNA PCR positive. NPV was defined as the probability that a specimen does not have a CD4:CD8 ratio ≥ 1 when the test was DNA PCR negative.

Comparisons of T cells between infected and uninfected infants were done using non-parametic equivalent of the T-test (Kruskal-Wallis test). *P *values less than 0.05 were considered statistically significant.

## Results

Of the 137 infant specimens tested using DNA PCR, 76 were HIV-1 positive and 61 were HIV-1 negative. The 76 PCR positive infants included 9 infants who had evidence of *in utero *transmission as determined by serial DNA PCR of birth and subsequent samples tested in longitudinal short course AZT MTCT studies (Zijenah et al, unpublished data).

### T cell subset of infected and uninfected infants

T lymphocyte subset profiles were performed for the 137 infants who had whole blood specimens for flow cytometry. The median age of these infants at specimen collection was 5.5 months (Interquartile Range [IQR]: 3–13) and 8.0 months (IQR: 4–14) for HIV infected and uninfected respectively (*p = 0.08*). As expected, the median CD4^+ ^cell counts of PCR negative infants were significantly higher than those of the PCR positive infants, *p < 0.001 *(Table 1). Inversely, the median CD8^+ ^cell counts were significantly higher among PCR positive infants than PCR negative infants, *p < 0.001 *(Table 1). The median CD4/CD8 ratio of the PCR positive infants (0.4, IQR: 0.3–0.6) was significantly lower than that of the PCR negative infants (1.8, IQR: 1.4–2.3), *p < 0.001*. The median %CD4 of PCR positive infants was also significantly lower than that of PCR negative infants, *p = < 0.01 *(Table 1).

**Table 1:** T cell subset profile of HIV-1 infected and uninfected infants

	**PCR Positive (n = 76)**	**PCR Negative (n = 61)**	***P *value^a^**
**Median age (months)**	5.5 (IQR: 3–13)	8 (IQR: 4–14)	0.08
**Median CD4^+^(cells/μL)**	521.5 (IQR: 323–805)	1356 (IQR: 916–1769)	<0.001
**Median CD8^+^(cells/μL)**	1302.5 (IQR: 829–2054)	799 (IQR: 471–1020)	<0.001
**Median CD4/CD8 ratio**	0.4 (IQR: 0.3–0.6)	1.8 (IQR: 1.4–2.3)	<0.001
**Median %CD4**	13.9 (IQR: 9.2–19.1)	29.9 (IQR: 25.3–34.5)	<0.001
**Median %CD8**	31.3 (IQR: 22.3–42.9)	18.4 (IQR: 14.2–21.5)	<0.001

Abbreviations: PCR, polymerase chain reaction; n, number tested; *P *value^a ^for statistical significance between group medians was estimated using the Kruskal-Wallis test.

### DNA PCR versus CD4/CD8 ratio

Seventy-five infants of the 76 PCR positive group (98.7%) had CD4/CD8 ratio <1 while 60 infants of the 61 who were PCR negative had CD4/CD8 ratio ≥ 1. All the 9 infants infected in utero had a CD4:CD8 ratio less than 1. Their median CD4/CD8 ratio was similar to that of the rest of the PCR positive babies (0.4; IQR: 0.3–0.6), albeit the numbers are too small for statistical significance considerations. The infant (aged 11 months at specimen collection) who was DNA PCR positive with a CD4/CD8 ratio of 1.1 was from the PACD study. The PCR negative infant (aged 4 months at specimen collection) with a CD4/CD8 ratio of 0.3 was also from the PACD cohort.

The overall sensitivity and specificity of the CD4/CD8 ratio were 98.7% (95% CI: 96.1–100 and 98.3% (95% Confidence Interval (CI): 95.1–100) respectively with PPV and NPV of 96.3% and 99.4% respectively and a test efficiency of 98.5% (95% CI: 96.5–100) (Table 2). Comparison of all the evaluated parameters between the 0–11 and 12–18 months infant age groups showed that the 95% CI overlap between the groups which implies no statistically significant difference in these two age groups.

**Table 2:** Evaluated parameters for CD4/CD8 ratio for the three infant age groups using DNA PCR as reference standard

	**0–18 months infant age group (n = 137)**	**0–11 months infant age group (n = 95)**	**12–18 months infant age group (n = 42)**
%Sensitivity	98.7 (CI: 96.1–100)	98.2 (CI: 94.7–100)	100 (CI: 100–100)
%Specificity	98.4 (CI: 95.2–100)	97.5 (CI: 92.7–100)	100 (CI: 100–100)
%PPV	96.4	94.6	100
%NPV	99.4	99.2	100
%TE	98.5 (CI: 96.5–100)	97.9 (CI: 95.0–100)	100 (CI: 100–100)

Abbreviations: n, number tested; NPV, negative predictive value; PPV, positive predictive value; TE, test efficiency.

## Discussion

In resource-poor countries, the major constraint in the use of DNA PCR for diagnosis of HIV-1 infection in infants under the age of 18 months is the cost of the equipment and the reagents. In addition, highly trained laboratory personnel and stringent quality assurance measures are needed to run this assay for routine diagnosis of HIV infection in infants.

In Zimbabwe, enzyme linked immunosorbent assay (ELISA) is routinely performed in both public and private laboratories for diagnosis of various infections including HIV-1. However, because of the transplacental transfer of maternal IgG antibodies, which may persist in infants for up to 18 months, ELISA is not suitable for diagnosis of HIV-1 infection in these infants. Therefore alternative methods are needed for this purpose. In addition, both public and private laboratories have flow cytometers or FACSCount machines for enumeration of T cell subset profile and automated haematological analysers for routine full blood counts with differential. With this equipment available in the country we evaluated the CD4/CD8 ratio as an alternative diagnostic test for infant HIV-1 infection.

In our investigation we used Epics XL Coulter flow cytometer equipped with System II software (Beckman Coulter, Miami Florida, USA) for the following reasons. With this instrument, CD4/CD8 ratios can be conveniently obtained. In addition, when used in double platform setting tandem with a haematological analyzer, the results also show absolute CD4^+ ^cell counts and both %CD4 and %CD8 values among lymphocytes. The %CD4 value among lymphocytes is the recommended parameter for analyzing pediatric samples, as absolute counts for infants are age sensitive and variable. A simpler single platform system such as the FACScount (Becton-Dickinson Immunocytometry Systems, San Jose, CA, USA), is not fully suited for pediatric work as it provides CD4^+^, CD8^+^, CD3^+ ^T lymphocyte counts and CD4/CD8 ratio but %CD4 are not available. Of note, %CD4 expressed among CD3^+ ^T lymphocytes, is a different parameter from the customary %CD4 expressed among lymphocytes. The %CD4 expressed among lymphocytes and not the %CD4 expressed among CD3^+ ^T lymphocytes is recommended for decision making to initiate ARV therapy in children under the age of 18 months [5].

In Zimbabwe, CD4^+ ^cell count, is less expensive than PCR and may provide additional information of value to the clinician with respect to prognosis, and the need for prophylaxis and treatment. Optimal flow cytometry for determination of T cell subset profile, offers the added advantage that CD4/CD8 ratio will determine the infection status of the infant while the % CD4 will provide information on decision-making for commencement of HAART.

Overall, the CD4/CD8 ratio had a ≥ 98% sensitivity for diagnosis of infant HIV-1 infection and a specificity ≥ 98%. Both sensitivity and specificity were 100% for infants in the 12–18 months age group. Interestingly, in parallel studies performed in 250 HIV-1 seropositive adults, 249 had a CD4/CD8 ratio of <1. The CD4/CD8 ratio of the one patient was 1 at enrollment and has remained so for over one year.

When interpreting our data, it is important to note that normal T cell subset values among African children differ from those of other populations [14-16]. A study in Guinea Bissau [16], reported that Guinean children under the age of 2 years had lower %CD4 and CD4/CD8 ratios and higher %CD8 when compared to their counterparts from developed countries. Interestingly, girls had higher CD4/CD8 ratios and lower %CD8 than boys. In our study, there were no statistically significant differences in absolute T cells, or percentages or CD4/CD8 ratio between boys and girls (Table 3 "refer to Additional file 1").

A very few studies in Africa have compared T cell subset profiles between HIV-1 infected and uninfected infants under the age of 2 years [14,17]. Moodley and colleagues in South Africa reported that the most marked changes in lymphocyte subset of HIV-1 infected children aged between 3 and 15 months were a lower %CD4 and higher %CD8 relative to uninfected infants [17]. In addition, CD4/CD8 ratio was a good predictor of poor clinical outcome at 3 months. The authors concluded that CD4/CD8 ratio and %CD4 among lymphocytes are reliable markers of HIV-1 infection in an African pediatric population. Furthermore a raised CD8^+ ^cell count rather than a CD4^+ ^cell count was a more specific prognostic marker of disease progression in HIV infected children.

Embree and colleagues, in Kenya, also reported that HIV-1 infected children had lower %CD4 and higher %CD8 by 3 months when compared to uninfected children [13]. The authors concluded that %CD4 and %CD8 among lymphocytes could be useful as an adjunct in HIV-1 diagnosis.

The two African studies mentioned above, have documented the clinical value of %CD4, CD4/CD8 ratio and CD8 counts in HIV-1 infection in infants.

In summary, the CD4/CD8 ratio may be useful in identifying infected infants while %CD4 will identify infants who may benefit from cotrimoxazole prophylaxis and/or initiation of HAART, and for management of HIV-infected infants in developing countries in general. We thus propose use of flow cytometry, where available, as a point of care diagnostic tool for ill infants admitted to hospitals with clinical symptoms suggestive of HIV infection and/or AIDS.

## Competing interest

The author(s) declare that they have no competing interests.

## Authors' contributions

LSZ designed the study, analyzed the data and drafted the manuscript. DAK, KJN, AB and GJ participated in the design of the study and preparation of the manuscript. SR conducted the statistical analysis and participated in the preparation of the manuscript. OT performed DNA PCR and analysed the data. AB and CG conducted flow cytometry and analysed the data, with the guidance of GJ. MN and PM participated in the collection of infant specimens and demographic data as well as preparation of the manuscript. All authors read and approved the final manuscript.

## Supplementary Material

Additional File 1 Table 3. Gender-based comparison of T cell subset profiles of HIV infected and uninfected babies. Click here for file
