# Supplementary material for: T lymphocytes among HIV-infected and -uninfected infants: CD4/CD8 ratio as a potential tool in diagnosis of infection in infants under the age of 2 years
Source: J Transl Med. 2005 Feb 1;3:6. doi: 10.1186/1479-5876-3-6 (PMC549040; doi:10.1186/1479-5876-3-6)
Supplement: Additional File 1 — Table 3. Gender-based comparison of T cell subset profiles of HIV infected and uninfected babies. [file 1479-5876-3-6-S1.doc]

Table 3. Gender-based comparison of T cell subset profiles of HIV infected and uninfected babies

|  | PCR Pos girls  (n = 30) | PCR Pos Boys  (n = 46) | *P value* (PCR Pos girls vs PCR Pos boys) | PCR Neg girls  (n = 33) | PCR Neg boys  (n = 28) | *P value* (PCR Neg girls vs PCR Neg boys) |
| --- | --- | --- | --- | --- | --- | --- |
| Median age (months) | 4 (IQR: 2-11) | 3 (IQR: 3.0-13.0 | 0.13 | 8 (IQR: 5.0-13.0) | 8 (IQR: 4.0-14.5) | 0.94 |
| Median CD4^+^ (cells/μL) | 547.5 (IQR: 358-825) | 508 (IQR: 316-794) | 0.94 | 1356 (IQR: 798-1771) | 1384 (IQR: 996-1672) | 0.64 |
| Median CD8^+^ (cells/μL) | 1186 (IQR: 875.5-1893) | 1389 (IQR: 814-2147) | 0.51 | 712 (IQR: 394-945) | 835(IQR: 634-1074) | 0.94 |
| Median CD4/CD8 ratio | 0.4 (IQR: 0.2-0.6) | 0.4 (IQR: 0.3-0.6) | 0.76 | 1.8 (IQR: 1.4-2.3) | 1.6 (IQR: 1.3-2.4) | 0.65 |
| Median  %CD4 | 14.2 (IQR: 9.5-17.2) | 13.5 (IQR: 9.0-20.0) | 0.99 | 29.8 (IQR: 25.5-35.0) | 30 (IQR: 25.3-33.7) | 0.90 |
| Median  %CD8 | 29.7 (IQR: 20.2-42.9) | 31.8 (IQR: 24.8-42.6) | 0.72 | 17.6 (IQR: 13.9-20.4) | 18.7 (IQR: 14.2-23.4) | 0.53 |

Abbreviations: PCR, polymerase chain reaction; Neg, negative, n, number tested; Pos, positive; *P* value^a^ for statistical significance between group medians was estimated using the Kruskal-Wallis test.
